# Shielding the Gut: Ghrelin and Ferrostatin-1’s Protective Role Against Sepsis-Induced Intestinal Ferroptosis

**DOI:** 10.3390/biomedicines13010077

**Published:** 2024-12-31

**Authors:** Qiliang Hou, Zhimin Dou, Lei Zhu, Bin Li

**Affiliations:** 1 Department of Critical Care Medicine, The First Hospital of Lanzhou University, Lanzhou 730000, China; 2 Department of Critical Care Medicine, The First Clinical Medical School, Lanzhou University, Lanzhou 730000, China; 3 National Key Laboratory of Critical Care Medicine, Lanzhou 730000, China

**Keywords:** sepsis, ghrelin, ferroptosis, intestinal barrier, systemic inflammatory response, oxidative stress

## Abstract

**Objective:** This study investigates the therapeutic efficacy of ghrelin in alleviating sepsis-induced intestinal damage, focusing on its potential to inhibit ferroptosis and protect intestinal barrier integrity. **Methods:** This study evaluates the therapeutic efficacy of intraperitoneal ghrelin (80 μg/kg) and Ferrostatin-1 (5 mg/kg) using a cecal ligation and puncture (CLP) model in C57BL/6 mice to determine their potential in alleviating sepsis-induced intestinal damage. The investigation focuses on the impacts of ghrelin and Ferrostatin-1 on bacterial load, intestinal morphology, systemic inflammation, oxidative stress, and ferroptosis markers. Our comprehensive methodology encompasses histopathological evaluations, cytokine profiling, oxidative stress assays, and detailed analyses of ferroptosis indicators to thoroughly assess the interventions’ efficacy. **Results:** Treatment with ghrelin significantly reduced bacterial proliferation, mitigated intestinal damage, and decreased systemic inflammation. Comparable outcomes were observed with Fer-1 treatment. Both interventions restored intestinal barrier functions, modulated inflammatory responses, and attenuated oxidative stress, indicating a suppression of the ferroptosis pathway. **Conclusion:** Ghrelin exhibits a protective role in sepsis-induced intestinal injury, likely through the inhibition of ferroptosis. This mechanism underscores ghrelin’s therapeutic potential in sepsis management, suggesting avenues for further clinical exploration.

## 1. Introduction

Sepsis is a severe and life-threatening condition that occurs when the body’s response to an infection spirals out of control, leading to widespread inflammation. This inflammation can trigger a chain reaction throughout the body, resulting in organ failure and, if not treated promptly, can lead to death. Sepsis is a major challenge in hospitals, especially in intensive care units, where it is one of the leading causes of death. It requires immediate medical attention to prevent severe complications or fatality [1]. Its lethality stems from a complex interplay of pathophysiological factors, with intestinal injury emerging as a pivotal, yet often underappreciated, component [2]. This introduction delves deeper into the dynamic interplay between sepsis, gut dysfunction, and a novel cell death pathway known as ferroptosis, highlighting the urgency of exploring ghrelin as a potential therapeutic shield against intestinal damage in sepsis.

The gastrointestinal tract, with its extensive mucosal surface and intricate immune crosstalk, is a front-line player in the systemic response to sepsis [3]. Disruption of its meticulously maintained barrier function during sepsis unleashes a host of detrimental events, including bacterial translocation and endotoxin spillage into the systemic circulation [4]. This not only exacerbates the inflammatory response but also underscores the critical connection between gut health and systemic inflammation [5,6]. The urgency for innovative therapeutic strategies focusing on intestinal protection and repair is more pronounced than ever, as this domain holds significant potential for altering the course of sepsis.

Recent scientific advancements have brought ferroptosis, an iron-dependent form of regulated cell death distinct from apoptosis and necrosis, into the limelight of sepsis research [7]. Characterized by the lethal accumulation of lipid peroxides, ferroptosis offers a unique perspective on cellular demise and has been implicated in a range of pathologies, including neurodegeneration, cancer, and ischemia-reperfusion injury [8,9]. Its emerging role in sepsis paints a complex picture of the pathophysiology underlying this condition and opens new avenues for therapeutic intervention.

Ghrelin, a multifaceted peptide hormone primarily secreted by the stomach, has gained attention for its role beyond appetite regulation and energy balance [7,8]. Exhibiting potent anti-inflammatory and antioxidative properties, ghrelin emerges as a promising candidate in combating the deleterious effects of sepsis, where unchecked inflammation and oxidative stress are rampant [10]. The capacity of ghrelin to modulate immune responses and potentially mitigate organ damage in septic conditions has sparked considerable interest in the scientific community, suggesting a new frontier in sepsis therapy.

This study embarks on an exploration of the intersection where ghrelin and ferroptosis converge in the context of sepsis-induced intestinal injury. We hypothesize that ghrelin’s role extends to acting as a potent ferroptosis inhibitor, thereby opening a novel therapeutic pathway to alleviate intestinal damage during sepsis. This investigation not only aims to deepen our understanding of ghrelin’s multifaceted biological roles but also seeks to pioneer new strategies for sepsis management, with a particular focus on gastrointestinal complications.

In addition to exploring ghrelin’s potential as a ferroptosis inhibitor, this study also considers the broader implications of such an interaction in the context of sepsis. By examining the mechanisms through which ghrelin influences ferroptosis and intestinal health, we aim to provide insights into the complex interplay between hormonal regulation, cell death pathways, and immune responses in septic conditions. This holistic approach is crucial for developing comprehensive strategies to combat sepsis, a condition that remains a significant challenge in modern medicine.

The intersection of sepsis, intestinal injury, and ferroptosis forms a complex puzzle with far-reaching clinical implications. This study aims to bridge the existing knowledge gap regarding ghrelin’s protective mechanisms in sepsis, focusing specifically on its potential role in inhibiting ferroptosis to alleviate intestinal injury. Through this endeavor, it aspires to make a substantial contribution to the ongoing battle against sepsis, offering new hope and potential therapeutic avenues for patients grappling with this life-threatening condition.

## 2. Methods

### 2.1. Sepsis and Intestinal Injury Mouse Model

Twenty-four 8-week-old male C57BL/6 mice, obtained from Chengdu Dashuo Biotech Co. Ltd. (Chengdu, China), were randomly divided into four groups (*n* = 6 per group): control, sepsis, sepsis + ghrelin, and sepsis + Ferrostatin-1 (Fer-1). The sepsis model was induced using cecal ligation and puncture (CLP), as previously described [11]. CLP is a widely accepted and clinically relevant model for mimicking human sepsis. In brief, anesthesia of the mice was conducted by the inhalation of isoflurane. It was induced at 2–3% for 2–3 min and maintained at 1–1.5%. After midline laparotomy, the cecum was exposed, ligated below the ileocecal valve, and punctured twice with an 18-gauge needle to allow the release of fecal content into the peritoneal cavity. The cecum was then returned to the abdominal cavity, and the incision was sutured. This procedure leads to polymicrobial peritonitis, which triggers a systemic inflammatory response characteristic of sepsis. The mice were monitored for signs of systemic illness, including hypothermia, lethargy, and piloerection, which confirmed the establishment of sepsis. Mortality and clinical scoring were used as additional indicators to verify the development and severity of the sepsis condition. Post-operative analgesia was provided with buprenorphine (0.1 mg/kg, subcutaneously) every 12 h for 24 h. The mice were monitored manually at 4 h intervals, including during the night, by two trained assessors who worked in shifts to ensure continuous observation over the 24 h post-intervention period. Each mouse was observed for clinical signs such as reduced mobility, piloerection, hypothermia, and lethargy, using a standardized scoring system derived from the Murine Sepsis Score [12]. Observations were cross-checked periodically during the study to minimize any scoring discrepancies. For the intervention, the sepsis + ghrelin and sepsis + Fer-1 groups received intraperitoneal injections of 80 μg/kg ghrelin and 5 mg/kg Fer-1, respectively. These doses were selected based on preliminary dose–response studies, demonstrating optimal therapeutic effects with minimal adverse outcomes. Previous studies, such as Zheng et al. (2019) [13] and Xiao et al. (2021) [14], further support these doses for mitigating sepsis-induced organ damage and dysfunction. The control and sepsis groups received equivalent volumes of saline. After 24 h, the mice were humanely euthanized using isoflurane inhalation, and blood and ileum tissues were collected for analysis.

All the animal procedures were conducted following the guidelines of the Institutional Animal Care and Use Committee (IACUC, Memphis, TN, USA) of Lanzhou University, and this study was approved by the ethics committee of the No. 1 Hospital of Lanzhou University (LDYYLL2024-42). The mice were housed in a controlled environment with a 12 h light/dark cycle, ad libitum access to food and water, and all efforts were made to minimize suffering; mice reaching the endpoint criteria were euthanized for humanitarian reasons. These were rapid weight loss of 15–20%; persistent temperature <34 °C or >39 °C with no return to normal; difficulty in breathing; and obvious signs of pain, such as spontaneous screaming, arching of the back, and thrashing behavior. None of the research animals reached these endpoints during the study.

### 2.2. Blood Culture

Blood samples were collected 24 h after the intervention. This timeline ensures accurate assessment of systemic effects post-treatment. Central blood collection was conducted via cardiac puncture and immediately diluted 10- or 100-fold with sterile saline. A 100 μL aliquot of each dilution was plated on blood agar plates (Biomeurix Inc., France) and incubated at 37 °C for 24 h. Colony-forming units (CFU/mL) were counted manually to assess bacterial load. All the blood culture experiments were conducted in triplicate, and the average CFU counts were recorded for each group.

### 2.3. Histological Analysis

Ileum tissues were harvested and fixed in 10% neutral-buffered formalin for 24 h, followed by dehydration, embedding in paraffin, and sectioning into 4 µm-thick slices. Hematoxylin and eosin (H&E) (Beyotime, Shanghai, China) staining was performed to assess tissue architecture and inflammatory infiltration. A blinded investigator evaluated the severity of intestinal injury using Chiu’s scoring system, which grades mucosal damage from 0 (normal) to 4 (severe destruction). The histological parameters assessed included villus length, crypt depth, epithelial shedding, and inflammatory cell infiltration. The scores were quantified, statistically analyzed, and presented as mean ± standard deviation.

For apoptosis detection, a TUNEL assay was conducted on the ileum sections using the In Situ Cell Death Detection Kit (Roche, Atlanta, GA, USA, 1168479590) following the manufacturer’s instructions. The sections were counterstained with DAPI for nuclear identification, and images were captured using a Pannoramic 250 Digital Slide Scanner (3DHISTECH). Apoptotic cells were quantified by counting TUNEL-positive cells per high-power field in five randomly selected fields per section [5].

### 2.4. Immunohistochemistry

Ileum sections were deparaffinized, rehydrated, and subjected to heat-induced epitope retrieval in citrate buffer (pH 6.0) for 15 min. The sections were blocked with 5% normal goat serum for 1 h at room temperature to prevent non-specific binding. Primary antibodies targeting ZO-1 (1:200), Occludin (1:200), Claudin-5 (1:100), ferritin (1:100), and transferrin (1:100) (ZSGB-BIO, Beijing, China) were applied overnight at 4 °C. After washing, the sections were incubated with HRP-conjugated secondary antibodies (ZSGB-BIO) for 1 h and visualized using diaminobenzidine (DAB, Hong Kong, China). The sections were counterstained with hematoxylin, and the staining intensity was quantified using ImageJ software (version 1.5) (NIH, Bethesda, MD, USA).

The immunoreactivity for tight junction proteins was measured as the percentage of DAB-positive area in five randomly selected fields per section, and statistical comparisons between groups were made.

### 2.5. TUNEL Assay

TUNEL staining, to detect apoptotic cells in the ileum sections, was performed using the In Situ Cell Death Detection Kit (1168479590, Roche), following the manufacturer’s protocol. Counterstaining with DAPI facilitated the identification of nuclei. Images were captured on a Pannoramic 250 Digital Slide Scanner (3DHISTECH).

### 2.6. Transmission Electron Microscopy (TEM)

Samples of the distal ileum were immediately fixed in 3% glutaraldehyde and post-fixed in 1% osmium tetroxide. After dehydration in a graded ethanol series, tissues were embedded in epoxy resin and sectioned into 70 nm ultra-thin slices. These sections were stained with uranyl acetate and lead citrate and examined under a JEOL 1010 electron microscope. Key features indicative of ferroptosis, including mitochondrial swelling, decreased cristae density, and membrane rupture, were analyzed and compared between groups.

### 2.7. Quantitative PCR

Total RNA was extracted from the ileum tissues using TRIzol reagent (Invitrogen, Carlsbad, CA, USA) and reverse transcribed into complementary DNA (cDNA) using the PrimeScript RT Reagent Kit (Takara, Osaka, Japan), according to the manufacturer’s protocol. Quantitative real-time PCR was conducted using the SYBR Green PCR Master Mix (Bio-Rad, Benicia, CA, USA) to measure the expression levels of IL-6, IL-1β, TNF-α, IL-22, and β-actin (internal control). The primers for IL-6, IL-1β, TNF-α, and β-actin were designed based on previously published references [15,16] while the primer for IL-22 was designed in-house and validated for specificity. All the primers used in this study are listed in Table 1.

### 2.8. Western Blotting

Ileum tissues were homogenized in RIPA buffer (Solarbio, Beijing, China) containing protease and phosphatase inhibitors. The protein concentration was determined using a BCA protein assay kit (A55861, Thermo Fisher, Waltham, MA, USA). Proteins (20 μg per lane) were separated by SDS-PAGE and transferred to PVDF membranes (Millipore, St. Louis, MO, USA). The membranes were blocked in 5% non-fat milk and incubated overnight with primary antibodies against 4-HNE (1:1000, MA5-27570, Thermo Fisher), GPX4 (1:1000, A1933, Abclonal, Woburn, MA, USA), ACSL4 (1:1000, A6826, Abclonal), PTGS2(1:1000, a1253, Abclonal), FTH1 (1:1000, A19544, Abclonal), SCL7A11(1:2000, DF12509, Affbiotech), and β-actin (loading control) (1:50000, AC026, Abclonal). After washing, the membranes were incubated with HRP-conjugated secondary antibodies (AS003, Abclonal; and S0001, Affbiotech, Redfern, Australia) and visualized using enhanced chemiluminescence (ECL, Bio-Rad). The bands were quantified using ImageJ software, and the relative protein expression levels were normalized to β-actin.

### 2.9. ELISA

Levels of DAO, FABP2, SOD, GSH, and MDA in ileum lysates were measured using specific ELISA kits (manufacturer listed in Table 2). These markers were chosen to assess intestinal integrity, oxidative stress, and inflammation. The ELISA assay was performed for the ileum samples following the instruction manual and the OD value was measured under 450 nm by a SpectraMAX Plus384 microplate reader (US Valley Molecular Instruments Co., Ltd., Shanghai, China).

### 2.10. ROS Detection

The ileum tissue was homogenized through a 200-mesh cell strainer. After two washes with PBS, the homogenate was centrifuged at 300× *g* for 5 min, and the cell pellet was collected for further use. Reactive Oxygen Species (ROS) levels were measured in ileum tissue homogenates using the Reactive Oxygen Species Assay Kit (S0033S, Beyotime, Shanghai, China). The homogenized tissues were stained with 2′,7′-dichlorofluorescein diacetate (DCFH-DA) and analyzed using a Beckman CytExpert Flow Cytometer. Fluorescence intensity was quantified, and ROS levels were expressed as mean fluorescence intensity (MFI).

### 2.11. Statistical Analysis

To ensure the appropriateness of our statistical tests, we first assessed the distribution of our data using the Shapiro–Wilk test. For data that conformed to a normal distribution (Shapiro–Wilk *p* > 0.05), analyses were performed using the mean ± standard deviation (SD), employing Student’s t-test for two-group comparisons and one-way ANOVA followed by Tukey’s post-hoc test for multiple group comparisons. For non-normally distributed data (Shapiro–Wilk *p* < 0.05), the results were reported as median and interquartile range (IQR), with the Mann–Whitney U test used for two-group comparisons and the Kruskal–Wallis test for multiple groups. Statistical significance was set at *p* < 0.05 for all the tests. All the analyses were conducted using SPSS software (Version 26.0).

## 3. Results

### 3.1. Ghrelin and Fer-1 Attenuate Bacteremia and Sepsis Severity

**Figure 1:**
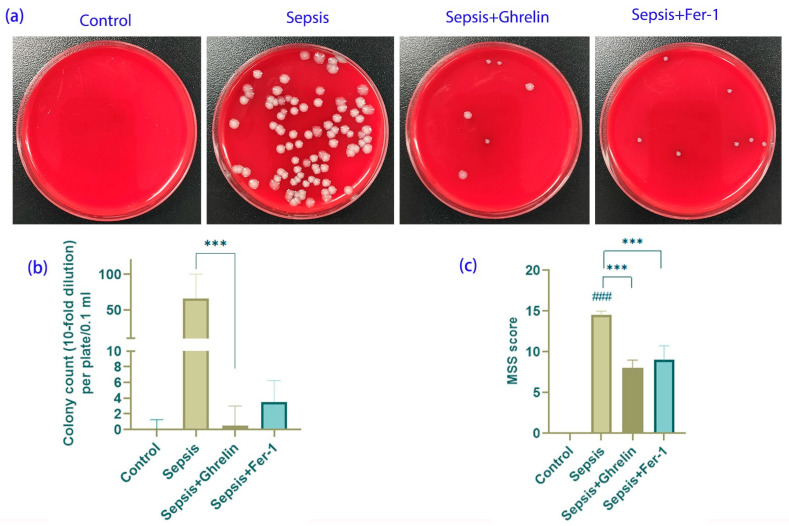
Effects of Ghrelin and Fer-1 on Bacterial Load and Sepsis Severity in C57BL/6 Mice. (**a**) Representative blood agar plates illustrating bacterial colonies from different treatment groups. The sepsis group shows a high number of colonies, indicative of bacteremia, which is substantially reduced in the ghrelin- and Fer-1-treated groups. (**b**) Quantification of bacterial colonies indicates a significant increase in CFU/mL in the sepsis group compared to the controls. Treatment with ghrelin or Fer-1 resulted in a marked reduction in CFU/mL, demonstrating their effectiveness in controlling bacterial proliferation. (**c**) The Murine Sepsis Score (MSS) assessing sepsis severity across groups. Sepsis induced a notable increase in MSS, which was significantly reduced by ghrelin and Fer-1 treatments, suggesting an amelioration of sepsis symptoms. (*** *p* < 0.001 compared to control group; ### *p* < 0.001 compared to sepsis group).

Bacteremia was assessed in a sepsis model using colony counts from blood agar cultures. The sepsis group exhibited a significant increase in bacterial colony formation. In contrast, treatment with ghrelin and Fer-1 markedly reduced bacterial counts, suggesting an ameliorative effect on LPS-induced bacteremia (Figure 1a,b). Furthermore, the Murine Sepsis Score (MSS), a measure of sepsis severity, was significantly lowered by both the ghrelin and Fer-1 treatments compared to the sepsis-only group, indicating an improvement in clinical outcomes (Figure 1c).

### 3.2. Protective Effects of Ghrelin and Fer-1 on Intestinal Morphology and Apoptosis

**Figure 2:**
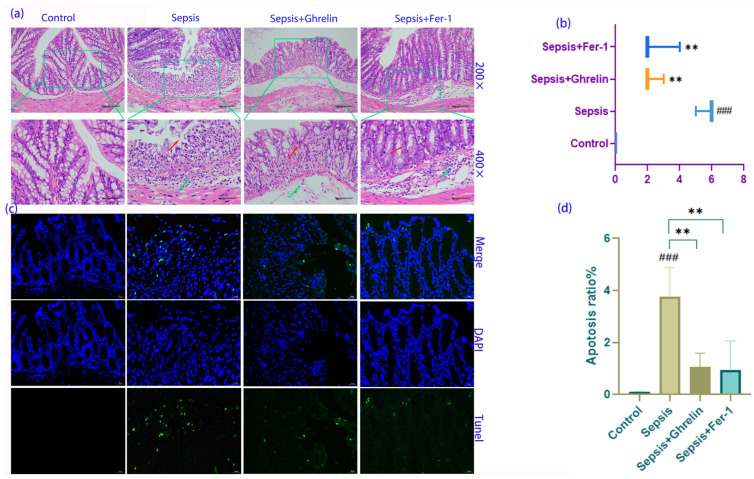
Assessment of Ghrelin and Fer-1 on Intestinal Morphology and Apoptosis in C57BL/6 Mice with Sepsis. (**a**) Histopathological analysis of ileum sections stained with hematoxylin and eosin (H&E). The control group shows normal architecture, while the sepsis group displays extensive mucosal damage (red arrow) and lymphomononuclear infiltration (green arrow). Ghrelin and Fer-1 treatment groups exhibit marked improvements with reduced mucosal damage and cellular infiltration. (**b**) Chiu’s score quantification indicates a significant increase in intestinal damage in the sepsis group compared to the control, with a substantial reduction observed in the ghrelin- and Fer-1-treated groups. (**c**) Fluorescent images from TUNEL assay depicting apoptotic cells in the intestinal wall. The sepsis group shows increased apoptosis (green fluorescence), which is mitigated in the ghrelin and Fer-1-treated groups. Scale bar: 20 μm. (**d**) Quantitative analysis of apoptosis ratio demonstrates a higher rate of cell death in the sepsis group, with a significant decrease following treatment with ghrelin and Fer-1. (** *p* < 0.01 compared to control group; ### *p* < 0.001 compared to sepsis group; ** *p* < 0.01 between indicated groups).

Histopathological examination revealed that sepsis led to extensive mucosal damage, epithelial shedding, and disruption of the intestinal glands, with a notable infiltration of lymphomononuclear cells. Treatment with ghrelin and Fer-1 markedly reduced tissue necrosis, inflammation, and glandular disruption, as evidenced by the H&E staining and the corresponding Chiu’s score (Figure 2a,b). The TUNEL assay further demonstrated that ghrelin and Fer-1 significantly decreased the number of apoptotic cells in the intestinal wall, indicating a protective effect against sepsis-induced cell death (Figure 2c,d).

### 3.3. Ghrelin and Fer-1 Enhance Intestinal Barrier Integrity and Modulate Inflammation

**Figure 3:**
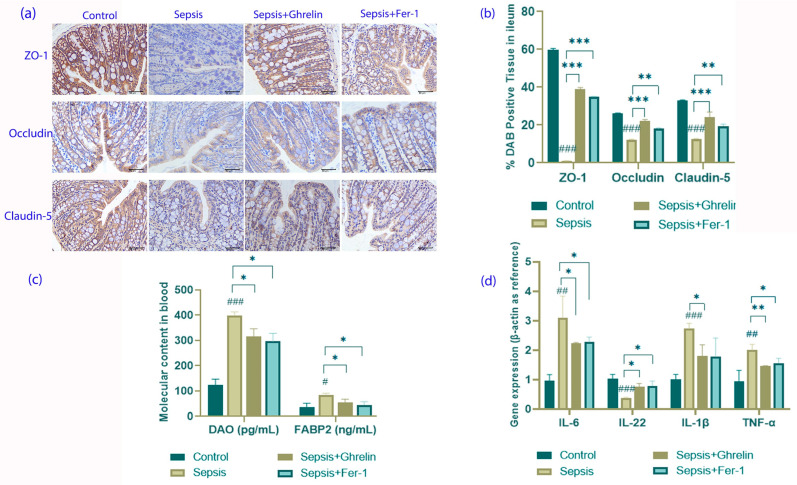
Evaluation of Effects of Ghrelin and Fer-1 on Intestinal Barrier Integrity and Inflammatory Response in Sepsis. (**a**) Immunohistochemical staining for tight junction proteins ZO-1, Occludin, and Claudin-5 in ileum sections. The control group displays robust staining, indicative of intact tight junctions. Sepsis reduces staining intensity, suggesting disrupted barrier integrity, which is notably restored by ghrelin and Fer-1 treatments. (**b**) Quantitative analysis of tight junction protein expression, showing a significant reduction in the sepsis group, with improvements observed in ghrelin- and Fer-1-treated mice. (**c**) Serum levels of intestinal barrier integrity markers DAO and FABP2. Elevated levels in the sepsis group are significantly reduced following treatment with ghrelin and Fer-1. (**d**) Gene expression of inflammatory cytokines IL-6, IL-22, IL-1β, and TNF-α. Sepsis induces a marked increase in pro-inflammatory cytokines and a decrease in IL-22, which are modulated towards normal levels in the treatment groups. (* *p* < 0.05, ** *p* < 0.01, *** *p* < 0.001 compared to controls; # *p* < 0.05, ## *p* < 0.01, ### *p* < 0.001 compared to sepsis group).

The integrity of the intestinal barrier, assessed by the expression of the tight junction proteins ZO-1, Occludin, and Claudin-5, was compromised in the sepsis group but was preserved by the ghrelin and Fer-1 treatment (Figure 3a,b). Additionally, ELISA analysis showed that both treatments significantly reduced the levels of DAO and FABP2, markers for intestinal barrier damage, suggesting a restoration of barrier function (Figure 3c). The cytokine profile analysis revealed that ghrelin and Fer-1 effectively normalized the expression of IL-6, IL-1β, TNF-α, and IL-22, which were dysregulated during sepsis, highlighting their anti-inflammatory properties (Figure 3d).

### 3.4. Ghrelin and Fer-1 Mitigate Oxidative Stress in Intestinal Cells

**Figure 4:**
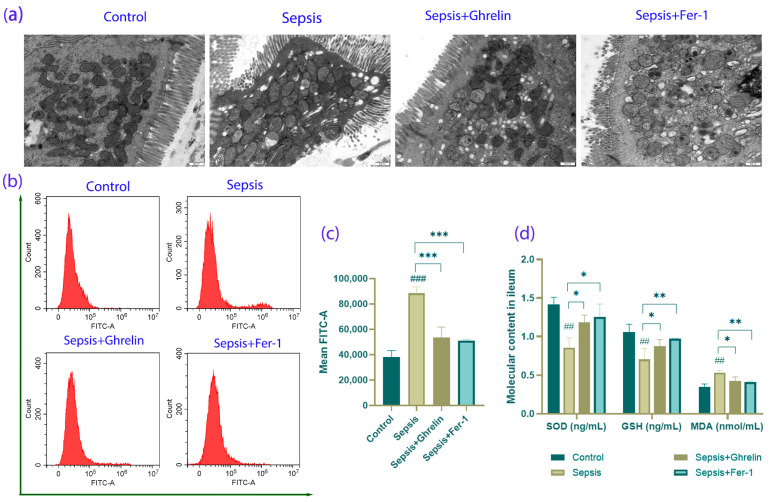
Analysis of Oxidative Stress and Mitochondrial Integrity in the Intestinal Cells of Sepsis Mice Treated with Ghrelin and Fer-1. (**a**) Transmission electron microscopy (TEM) images showing intestinal epithelial cell ultrastructure. The control group maintains normal microvilli and mitochondrial morphology. Sepsis induces significant alterations including sparse microvilli and mitochondrial swelling, which are notably improved by ghrelin and Fer-1 treatment. Scale bar: 600 nm. (**b**) Flow cytometry histograms representing Reactive Oxygen Species (ROS) levels as indicated by FITC-A fluorescence. Sepsis significantly increases ROS production, while ghrelin and Fer-1 treatments are associated with lower fluorescence intensity, indicating reduced ROS levels. (**c**) Quantitative analysis of mean FITC-A fluorescence confirms the increase in ROS in septic mice and the effective reduction in ROS by both ghrelin and Fer-1 treatments. (**d**) Levels of oxidative stress markers SOD (Superoxide Dismutase), GSH (Glutathione), and MDA (Malondialdehyde) in ileum tissue. Sepsis alters these biomarkers indicative of oxidative stress, which is modulated by treatment with ghrelin and Fer-1, reflecting improved oxidative homeostasis. (*** *p* < 0.001 compared to controls; * *p* < 0.05, ** *p* < 0.01 compared to sepsis; ## *p* < 0.01, ### *p* < 0.001 compared to controls).

Transmission electron microscopy provided insight into the structural integrity of the intestinal cells. In the sepsis group, there was evidence of sparse microvilli, condensed cytoplasm and nucleus, mitochondrial swelling, and disrupted tight junctions. Remarkably, the ghrelin and Fer-1 treatments preserved the density of the microvilli and mitochondrial structure, suggesting reduced oxidative stress (Figure 4a). Flow cytometry analysis supported this, showing that ghrelin and Fer-1 significantly lowered ROS levels (Figure 4b,c). Additionally, the antioxidant markers SOD and GSH and the lipid peroxidation marker MDA were modulated towards control levels in the treatment groups, further confirming the antioxidant effects of the treatments (Figure 4d).

### 3.5. Inhibition of Ferroptosis by Ghrelin and Fer-1 in Sepsis

**Figure 5:**
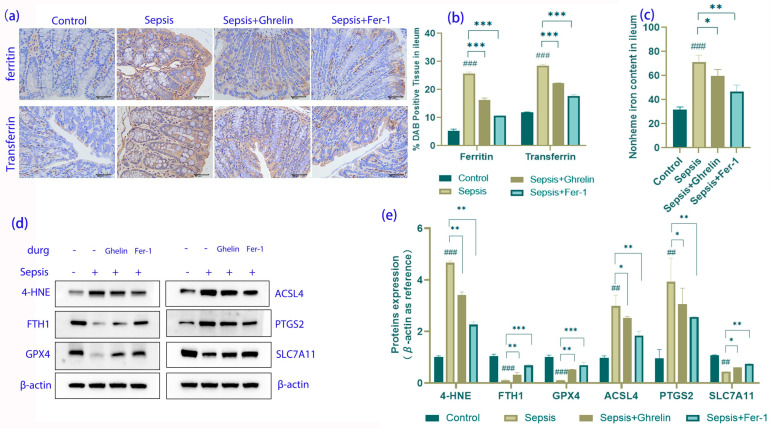
Impact of Ghrelin and Fer-1 on Ferroptosis Markers in Intestinal Cells during Sepsis. (**a**) Immunohistochemistry for ferroptosis-associated proteins ferritin and transferrin in ileum tissue. Staining intensity is increased in sepsis, indicating elevated iron storage and transport, which is mitigated by ghrelin and Fer-1 treatments. (**b**) Quantitative analysis of DAB positive tissue area showing protein expression of ferritin and transferrin. There is a notable elevation in the sepsis group, with both treatments effectively reducing expression levels. (**c**) Quantification of nonheme iron in the ileum, with an increase observed in septic mice, and a reduction following treatment with ghrelin and Fer-1, suggesting a decrease in free iron contributing to ferroptosis. (**d**) Western blot analysis of ferroptosis-related proteins including 4-HNE, FTH1, GPX4, ACSL4, PTGS2, and SLC7A11. Altered expression patterns in sepsis are partially normalized by ghrelin and Fer-1 treatment. (**e**) Densitometric quantification of the protein bands normalized to β-actin, confirming the modulation of ferroptosis markers by treatments, indicative of a protective effect against ferroptosis. (*** *p* < 0.001, ** *p* < 0.01, * *p* < 0.05 compared to controls; ### *p* < 0.001, ## *p* < 0.01 compared to sepsis group).

Immunohistochemical staining showed that sepsis significantly upregulated the levels of ferritin and transferrin, indicating increased iron storage and transport associated with ferroptosis. However, both the ghrelin and Fer-1 treatments reduced the expression of these proteins (Figure 5a,b). The nonheme iron content in the ileum followed a similar pattern, with both treatments decreasing iron levels relative to the sepsis group (Figure 5c). Western blot analysis of ferroptosis-related proteins revealed that sepsis altered the expression of 4-HNE, FTH1, GPX4, ACSL4, PTGS2, and SLC7A11, which was reversed by ghrelin and Fer-1, indicating their role in inhibiting ferroptosis (Figure 5d,e).

## 4. Discussion

The multifaceted nature of sepsis, with its propensity to cause multiple organ dysfunction syndrome (MODS), represents a formidable challenge in modern medicine [17]. The pathogenesis of sepsis is complex, involving an orchestrated response that includes both immune activation and suppression, leading to tissue damage and organ failure [18]. Our study utilized the cecal ligation and puncture model to induce sepsis, which is considered the gold standard for its clinical relevance as it closely mimics the progression of human sepsis [15]. The subsequent bacteremia and inflammatory response observed in our model set the stage for assessing the therapeutic potential of ghrelin and Fer-1.

### 4.1. Ghrelin’s Multidimensional Role in Sepsis Management

Ghrelin, traditionally known for its growth hormone-releasing activity, has emerged as a molecule of interest due to its anti-inflammatory and tissue-protective effects [16]. Ghrelin has been extensively studied for its multifaceted protective roles in sepsis, with multiple interpretative models shedding light on its mechanisms of action. Wan et al. (2016) demonstrated that ghrelin protects the small intestinal epithelium from sepsis-induced injury by enhancing autophagy in intestinal epithelial cells, a process crucial for maintaining cellular homeostasis and reducing inflammatory responses [19]. This study emphasized the upregulation of autophagy-related proteins, such as LC3, Atg7, and Beclin 1, mediated by ghrelin through AMPK-dependent pathways, which also mitigates mitochondrial dysfunction and oxidative stress, key drivers of sepsis-induced organ damage. Furthermore, ghrelin has been shown to modulate the immune response by inhibiting NF-κB activation, thereby reducing pro-inflammatory cytokines such as TNF-α, IL-1β, and IL-6. Other studies, such as Wu et al. (2009) [20], have highlighted ghrelin’s ability to preserve gut barrier integrity by improving microcirculatory blood flow and preventing bacterial translocation, which are critical in systemic sepsis progression. Additionally, its orexigenic and anti-apoptotic properties may further contribute to improved survival outcomes in septic models. Moreover, alternate models as discussed in the referenced article [15,17] suggest that ghrelin’s effects on modulating metabolic pathways and enhancing immune cell functionality play significant roles in its protective mechanisms against sepsis. These findings support the hypothesis that ghrelin’s protective effects are mediated through a combination of anti-inflammatory, antioxidative, and pro-autophagic mechanisms. However, the interplay between these mechanisms and their relative contributions in different septic models remain to be fully elucidated, underscoring the need for future studies to explore ghrelin’s systemic effects across various organ systems and septic etiologies.

### 4.2. Intestinal Barrier Integrity: A Keystone in Sepsis Pathophysiology

The maintenance of the intestinal barrier is critical in the prevention of sepsis, as it shields the internal milieu from pathogenic microbial translocation [21,22]. In our study, the marked increase in tight junction proteins ZO-1, Occludin, and Claudin-5 in response to ghrelin treatment underscores its potential to fortify this barrier. This finding is especially relevant given the increasing recognition of gut microbiota and intestinal barrier integrity in health and disease. The decrease in serum DAO and FABP2 levels further supports ghrelin’s barrier-strengthening effects [22], which could have significant implications for preventing the progression from local infection to systemic sepsis.

The novel findings of our research emphasize ghrelin’s comprehensive role in modulating the immune response during sepsis, a critical aspect that has been underexplored. We have identified specific mechanisms through which ghrelin interacts with various immune cells via the growth hormone secretagogue receptor (GHS-R1a) [23,24], facilitating a nuanced influence over both the innate and adaptive immune systems. Notably, our results highlight ghrelin’s capacity to mediate macrophage polarization, shifting their phenotype from pro-inflammatory (M1) to anti-inflammatory (M2). This shift is crucial for reducing the production of inflammatory cytokines such as TNF-α, IL-6, and IL-1β, while promoting the secretion of anti-inflammatory cytokines like IL-10. Such modulation is vital for attenuating the typically exaggerated inflammatory response seen in sepsis and suggests a therapeutic target for mitigating systemic inflammation [25,26]. Moreover, our investigation into ghrelin’s effects on neutrophil function sheds light on its ability to reduce neutrophil extracellular trap (NET) formation and limit neutrophil infiltration into tissues. These actions are significant as they address critical components of disease progression in sepsis, where neutrophil dysfunction can exacerbate the condition. Our study also ventures into the emerging area of ghrelin’s influence on T-cell responses, demonstrating its potential to enhance regulatory T-cell functions and modulate the balance between regulatory and effector T-cells. This balance is essential for controlling the immune response during sepsis and suggests that ghrelin could help restore immune homeostasis and prevent sepsis-induced immunosuppression, possibly through interactions with immune checkpoint pathways such as PD-1/PD-L1 [27,28,29].

The comprehensive analysis of cytokine profiles in our study further substantiates ghrelin’s immunomodulatory role, with observed reductions in pro-inflammatory cytokines and enhancements in anti-inflammatory cytokine IL-22. These findings not only support ghrelin’s therapeutic potential but also open up avenues for future research to dissect the specific immune cell signaling pathways involved. Particularly, the roles of NF-κB as a central regulator of inflammation [30] and the JAK/STAT pathways crucial for cytokine signaling are areas ripe for further exploration. Additionally, the interplay between ghrelin and autophagy in immune cells introduces a layer of complexity that merits in-depth study, given autophagy’s protective role in cellular stress and inflammation, which could amplify ghrelin’s immunomodulatory effects.

### 4.3. Oxidative Stress and Ferroptosis: New Horizons in Sepsis Therapy

The pathophysiology of sepsis is intricately linked with oxidative stress, which exacerbates cellular injury and contributes to organ dysfunction [31]. Our study enhances the growing body of evidence implicating ferroptosis, a regulated form of iron-dependent cell death, in the pathogenesis of sepsis. By demonstrating the mitigation of oxidative stress markers and the preservation of mitochondrial integrity with ghrelin and Fer-1 treatment, we provide novel insights into potential therapeutic targets [24,26,28,30,32]. The observed reductions in nonheme iron content and alterations in ferroptosis-related proteins, particularly GPX4 and ACSL4, underscore the significant role of iron metabolism and ROS management in sepsis treatment strategies [32,33]. These findings not only highlight the modulation of these pathways by ghrelin and Fer-1 but also suggest a complex regulatory network involving iron regulatory proteins such as ferritin and transferrin. Such interactions are critical as they potentially stabilize cellular redox homeostasis and limit oxidative damage during sepsis.

Furthermore, the pathways involving iron homeostasis and mitochondrial oxidative stress are critical to understanding how ghrelin exerts its protective effects in sepsis. The decrease in iron content specifically targets the pro-oxidative conditions prevalent in sepsis, potentially reducing the risk of ferroptosis and improving survival outcomes. This aligns with emerging research suggesting that targeting mitochondrial dysfunction and enhancing the body’s antioxidant defenses can significantly impact sepsis outcomes [33,34,35].

### 4.4. Clinical Implications and Future Research Directions

The therapeutic implications of our findings are substantial, suggesting that ghrelin or ghrelin analogs could be explored as adjunctive treatments in sepsis. The translation of these preclinical findings to clinical settings necessitates rigorous randomized controlled trials to assess the efficacy and safety of ghrelin in sepsis patients. Furthermore, the potential for combining ghrelin with other sepsis treatments, such as antibiotics and fluid resuscitation, offers a multipronged approach to this complex condition.

While this study provides valuable insights into the protective role of ghrelin in sepsis-induced intestinal ferroptosis, several limitations should be acknowledged. First, the study was conducted using an animal model, and although the CLP method is a well-established model for sepsis, the findings may not fully translate to human sepsis due to differences in immune response and physiology. Second, while the ghrelin and Fer-1 treatments showed promising protective effects, the long-term outcomes of these treatments were not evaluated, and the dose–response relationship for ghrelin requires further investigation. Additionally, although we provided substantial evidence of ferroptosis inhibition, the mechanistic pathways involved in ghrelin’s interaction with ferroptosis-related markers (such as GPX4, ACSL4, and PTGS2) warrant more in-depth molecular exploration. Lastly, we did not explore the potential effects of ghrelin on other organs affected by sepsis, which could provide a more holistic understanding of its therapeutic potential.

More comprehensive understanding of the specific molecular interactions between ghrelin and iron homeostasis, as well as its antioxidant effects, is crucial. These mechanisms may involve complex pathways, including ghrelin’s influence on iron regulatory proteins (e.g., ferritin, transferrin) and its potential to modulate mitochondrial function and ROS production. Future studies focusing on these detailed pathways, including investigating signaling cascades such as Nrf2/HO-1 and iron transporters that may mediate ghrelin’s role in ferroptosis inhibition, the exploration of ghrelin’s impact on the gut microbiome, its role in immune cell regulation, and its potential to modulate autophagy, will provide a more comprehensive understanding of its therapeutic mechanisms. Additionally, the dose–response relationship, optimal timing for administration, and long-term outcomes of ghrelin treatment in sepsis merit further study.

### 4.5. Limitation

While the CLP model is widely regarded as the gold standard for studying polymicrobial sepsis, it is important to acknowledge its specific characteristics and potential limitations. The CLP model effectively mimics key features of human sepsis, including systemic inflammation, bacterial dissemination, and multi-organ dysfunction, making it a valid substitute for studying general sepsis mechanisms. However, as the model initiates sepsis through intestinal perforation, it is particularly relevant for studying sepsis with an intestinal origin. Despite its robustness, the CLP model may not fully capture the complexity of human septic conditions, such as specific host–pathogen interactions, variations in immune responses, or co-morbidities often seen in clinical settings. To enhance the translational potential of our findings, future studies could employ complementary models, such as endotoxemia or pneumonia-induced sepsis, to address these limitations and broaden the scope of investigation into sepsis pathophysiology.

## 5. Conclusions

Our study has illuminated the potential of ghrelin as a multifaceted therapeutic agent in sepsis, particularly in mitigating intestinal injury and systemic inflammation. By attenuating bacterial proliferation, reducing the severity of sepsis as indicated by MSS scores, and improving intestinal morphology, ghrelin demonstrates significant promise. Its comparison with Fer-1, a known ferroptosis inhibitor, suggests that ghrelin’s beneficial effects might be mediated through the inhibition of ferroptosis, providing an innovative angle for sepsis treatment strategies. The restoration of intestinal barrier integrity, as evidenced by the upregulation of critical tight junction proteins and the reduction in serum DAO and FABP2 levels, further underscores ghrelin’s protective role against the leakage of bacterial and toxic substances into the systemic circulation.

Additionally, ghrelin and Fer-1’s capability to modulate oxidative stress and preserve mitochondrial integrity opens new therapeutic avenues, especially considering the pivotal role of oxidative damage in sepsis pathophysiology. These findings, together with the modulation of inflammatory and ferroptosis-related markers, offer a comprehensive approach to tackling the complex nature of sepsis. Future research should aim to unravel the precise molecular mechanisms by which ghrelin exerts these effects and to determine the clinical efficacy of ghrelin through controlled trials. Ultimately, our research adds to the growing body of evidence that supports the exploration of ghrelin as a novel therapeutic intervention for sepsis, with the potential to significantly enhance patient outcomes.

## Figures and Tables

**Table 1:** Primers for qPCR assay.

Genes	Forward Primer	Reverse Primer
β-actin	CTACCTCATGAAGATCCTGACC	CACAGCTTCTCTTTGATGTCAC
IL-1β	CACTACAGGCTCCGAGATGAACAAC	TGTCGTTGCTTGGTTCTCCTTGTAC
IL-6	CTCCCAACAGACCTGTCTATAC	CCATTGCACAACTCTTTTCTCA
IL-22	GCAGATAACAACACAGATGTCC	GTCTTCCAGGGTGAAGTTGAG
TNF-α	ATGTCTCAGCCTCTTCTCATTC	GCTTGTCACTCGAATTTTGAGA

**Table 2:** ELISA kits and manufacturer.

ELISA Kits	Manufacturer
Mouse DAO ELISA KIT(ZC-38123)	Shanghai ZCi Bio Co., Ltd. (Shanghai, China)
Mouse FABP2 ELISA KIT (ZC-39010)	Shanghai ZCi Bio Co., Ltd. (Shanghai, China)
Mouse SOD ELISA KIT (ZC-38236)	Shanghai ZCi Bio Co., Ltd. (Shanghai, China)
Mouse GSH ELISA KIT (ZC-38228)	Shanghai ZCi Bio Co., Ltd. (Shanghai, China)
Mouse MDA ELISA KIT (ZC-55718-J)	Shanghai ZCi Bio Co., Ltd. (Shanghai, China)

## Data Availability

The data presented in this study are available on request from the corresponding author due to privacy.
